# Action to achieve smoke-free homes- an exploration of experts' views

**DOI:** 10.1186/1471-2458-9-112

**Published:** 2009-04-22

**Authors:** Deborah Ritchie, Amanda Amos, Richard Phillips, Sarah Cunningham-Burley, Claudia Martin

**Affiliations:** 1 Nursing Studies, School of Health in Social Science, University of Edinburgh, Medical School, Teviot Place, Edinburgh, EH8 9AG, UK; 2 Public Health Sciences, Centre for Population Health Sciences, University of Edinburgh, Medical School, Teviot Place, Edinburgh, EH8 9AG, UK; 3 Scottish Centre for Social Research, 73 Lothian Road, Edinburgh, EH3 9AW, UK

## Abstract

**Background:**

Smoking in the home is the major cause of exposure to second-hand smoke in children in the UK, particularly those living in low income households which have fewer restrictions on smoking in the home. Reducing children's exposure to second-hand smoke is an important public health and inequalities issue. Drawing on findings from a qualitative Scottish study, this paper identifies key issues and challenges that need to be considered when developing action to promote smoke-free homes at the national and local level.

**Methods:**

Two panels of tobacco control experts (local and national) from Scotland considered the implications of the findings from a qualitative study of smokers and non-smokers (who were interviewed about smoking in the home), for future action on reducing smoking in the home.

**Results:**

Several key themes emerged through the expert panel discussions. These related to: improving knowledge about SHS among carers and professionals; the goal and approach of future interventions (incremental/harm reduction or total restrictions); the complexity of the interventions; and issues around protecting children.

**Conclusion:**

The expert panels were very aware of the sensitivities around the boundary between the 'private' home and public health interventions; but also the lack of evidence on the relative effectiveness of specific individual and community approaches on increasing restrictions on smoking in the home. Future action on smoke-free homes needs to consider and address these complexities. In particular health professionals and other key stakeholders need appropriate training on the issues around smoking in the home and how to address these, as well as for more research to evaluate interventions and develop a more robust evidence base to inform effective action on this issue.

## Background

Smoking is a major cause of inequalities in health in Scotland [1]. In 2003 41% of men and 39% of women in semi-routine and routine occupations smoked cigarettes compared to 17% of men and 16% of women in professional and managerial occupations [2]. There are even greater differences at the local level with over 50% of adults being smokers in the most deprived areas [3]. Not only is smoking more prevalent in these communities but, prior to the introduction in March 2006 of smoke-free legislation in Scotland, bars, pubs and other workplaces in areas of socio-economic disadvantage were less likely to have smoking policies and more likely to permit smoking than in affluent communities [4,5]. In 2003 over 80% of children aged 8 to 15 years in Scotland reported being exposed to SHS, most commonly in their own homes [2]. 40% lived in a home with at least one smoker and this was highest among low income households.

Previous studies, for example in Ireland and New Zealand, have found that the introduction of smoke-free legislation is associated with reported increases in home smoking restrictions [6,7]. The Scottish smoke-free legislation has been successful in reducing children's and adults' exposure to second-hand smoke (SHS) [8,9]. However, the evidence from Scotland showed that there were only significant reductions in children's exposure to SHS where neither parent or only the father smoked [8]. The home and the car are two key sites of SHS exposure for children [10]. Many children often do not have the personal power to complain or to protect themselves from exposure to SHS [11,12], despite the evidence linking SHS exposure to the cause and exacerbation of a number of childhood illnesses[10,13]. There is therefore an increasing focus at national and local levels on action to protect children by enabling more homes to become smoke-free [14].

Reducing children's exposure to SHS in the home is an important public health and inequalities issue. A recent British Medical Association (BMA) report [10] highlighted the clear evidence linking SHS exposure to the exacerbation of illnesses, such as asthma and middle ear infections, with consequent poor school attendance and attainment, and increased hospital admissions. These significant illness experienced by many children exposed to SHS are further exacerbated by existing inequalities and impact upon the life chances of many children [10]. Exposure is linked to parents' and other carers' poor knowledge of the relationship between exposure to SHS and the specific health risks for children [15]. Qualitative research carried out in Scotland following the introduction of the smoke-free legislation found that even though there had been a Scottish media campaign on the risks of SHS, adults' understanding of the health risks was often limited and/or confused [16].

Health professionals are in a unique position to encourage smoke-free homes for children. There is however little conclusive evidence that health professionals' interventions are effective [10,17]. A Cochrane review of 18 RCTs designed to reduce parents' smoking prevalence or reduce children's level of exposure found insufficient evidence to recommend one intervention strategy over another [18]. Three more recent US studies involving health professionals have showed promise. Greenberg and colleagues [19] found that nurses using self help materials and counselling parents in their home reduced home exposure, but did not increase smoking cessation. Hovell et al [20] used counselling and feedback of the child's pulmonary function for parents of asthmatic children with some success. More recently Winickoff et al [21] demonstrated in an outpatient paediatric clinic that it was feasible to encourage quit attempts with counselling and NRT and reduce consumption in the home and the car. However, another study [22] found that the rates of counselling parents about smoke-free homes were extremely low. More recently a US review [23] stressed the importance of effective SHS interventions for 'medically at risk' children. It concluded that intensive multiple level interventions, including interventions in a medical setting, that reduce children's exposure to SHS and those aimed at encouraging parents to quit do have some success.

Two studies involving disadvantaged parents in Liverpool and Australia have generated insights about a range of social, physical, psychosocial and economic factors parents perceive as barriers to reducing their children's SHS exposure in the home [24-27]. These include difficulties associated with the supervision of children, lack of appropriate outdoor space to smoke, a desire to smoke in comfort and/or privacy, concerns about the negative reactions of family and friends, and the lack of support from partners and friends, as well as nicotine dependence. In addition the Liverpool study found that while mothers thought that babies should not be exposed to SHS, and reported strategies to deal with this; few had continued these into infancy [25,26].

Increasingly community-based initiatives are emerging throughout the UK aimed at encouraging more smoke-free homes. Often focusing in particular on parents in disadvantaged communities, they use one of two different approaches. The first takes an incremental, staged or 'harm reduction' approach, such as the Breathe Easy initiative in Glasgow and the West Yorkshire Smoking and Health Smoke Free Homes Project which involves parents pledging or promising to make their home smoke-free (Gold) or, if they feel that this is not possible at present, to have no smoking in one room (Silver) with the aim of eventually becoming totally smoke- free. Similarly Salford's Smoke-free Homes Scheme offers three awards: Gold (totally smoke-free), Silver (not smoking in the presence of children, smoking limited to one well ventilated room) and Bronze (not smoking in the presence of children or other non-smokers) . In contrast the second approach promotes only totally smoke-free homes such as the East Sussex Smoke Free Homes Campaign . However, there is little or no evidence on which is the most effective approach or the experts' views on these approaches and the issues that need to be considered.

In order to help inform the development of effective action on this issue, this paper explores the views of people working in tobacco control, at the local and national level in Scotland, about the potential for and feasibility of creating smoke-free homes. Two expert panels were convened to discuss the findings from a qualitative study of smoking in the home conducted by the authors shortly after the smoke-free legislation in Scotland [16]. The primary study found that most people reported that they restricted smoking in their homes – either partial or full restrictions. Children and grandchildren were important considerations in the development and modification of smoking restrictions in the home. However, the strategies that people used to regulate smoking in their homes and cars were often complex and fluid, sometimes involving measures, such as opening windows or only smoking in one room, that were likely to be ineffective in reducing SHS exposure [16,28,29].

## Methods

### Study Design

The study used qualitative methods to examine the behaviours and attitudes associated with smoking in the home. The study involved three phases. Phase 1 (June–September 2006) involved 50 semi-structured interviews, using a topic guide and day grid recording participants' smoking patterns and/or daily exposure to SHS, with individuals recruited from across Scotland. Respondents were selected to encompass factors which might influence home smoking restrictions and responses to the smoking legislation including: household composition (with and without children at home, smokers living with non-smokers, non-smokers living with smokers, smokers living with smokers, couples with the same or different smoking status), socio-economic status, rural/urban location, gender. Phase 2 (December 2006–January 2007) focused on 9 household cases, selected on theoretical grounds, where additional household members were interviewed. A detailed description of Phases one and two are reported in Phillips et al 2007 [16].

In the final phase (June 2007), which is the focus of this paper, two panels of experts with experience of tobacco control and community development considered the study findings. The panel members were recruited purposively from networks within Scotland and included people who worked in tobacco control as part or the whole of their role, at the national and local level (Table 1). This included some people who were working in community smoke-free homes initiatives. The panels were convened to discuss the findings of the primary study [16] and to derive from their expert knowledge the implications for the development of interventions on reducing SHS exposure in the home. The panels aimed, by drawing upon the shared expertise and insights of the two groups, to generate a partnership process of analysis. Participants were sent in advance a summary of the study findings (Table 2) and key questions for discussion (Table 3).

**Table 1:** Membership of Expert Panels

***City 1 Panel***
Lecturer/Health Visitor (1)
Health promotion specialist (2)
National tobacco control alliance (3)
National Public health agency (4)
Community health partnership (5)
Voluntary organization for community smoking initiatives (9)
Smoking cessation co-ordinator (7)

***City 2 Panel***
Local health partnership (12)
Voluntary organization for community smoking initiatives (9)
Community health partnership (10)
Regional tobacco policy manager (11)
National Public health agency (12)
National Public health agency (13)
Public health practitioner-smoking (14)

**Table 2:** Key primary study findings for the expert panels to consider

1. Passive smoking was a well recognised term but people had varied understandings of the risks of SHS, with a few rejecting evidence of such risks. Children were generally perceived as vulnerable to the effects of SHS.
2. Most reported they restricted smoking in their homes but this varied in extent and likely effectiveness. Spatial, health, relational and aesthetic factors were influential with a key consideration being protecting children and grandchildren.

3. Other important underlying factors were: the meaning of the home as somewhere private, social identity (being hospitable and not anti-smoker), and moral identity (being a caring parent or grandparent).

4. There are more reported restrictions on smoking in their cars, which is seen as being a more confined space.

5. People had diverse views on the Scottish smoke-free legislation. Few thought it had influenced their home restrictions or smoking in the home.

6. Awareness of the risks of SHS, despite ambivalence about health messages and the fluidity of smoking restrictions, provide clear opportunities for public health initiatives

**Table 3:** Key questions for the expert panels

**Reflect upon the findings and consider the following questions:**
1. What is relevant for you in your own area of expertise?
2. What is new? What is of particular interest?
3. How could you use the findings?
4. What needs to be done for policy?
5. What needs to be done for health promotion practice?
6. What are the enablers and opportunities?
7. What problems/barriers or ethical issues do you envisage?

Both panels were facilitated by two members of the study team. Each panel member introduced themselves and described the experience and expertise that they brought to the group. This was followed by a presentation of the main study findings and a group discussion of the findings and key questions for an hour (Table 2, &3). Each participant then worked individually for 15 minutes identifying a key personal learning point; a priority action for practice; a priority action for policy; and considered how they would use the findings within their own area of practice. Finally the group shared their individual summary points and with the facilitators negotiated a consensus summary of the priorities for action and debate.

### Analysis

The discussions of the expert panels were tape recorded and transcribed. In addition detailed flipcharts recorded the main reflections during the group discussions. Reflective field-notes of the discussions were also taken by both facilitators. The data were analysed inductively, involving the whole research team as an interpretive community. An interpretive research community engages in a process of analysis, as part of a team based approach to qualitative analysis, by working reflexively as a team [30].

The data were interrogated systematically, by firstly identifying emergent themes and issues, and then moving from this descriptive thematic coding to the analytical coding. The analytical coding involved making comparisons across the themes and within themes in order to explore the more explanatory concepts; and in order to ensure that different views and positions within themes were considered, such as different views regarding child protection. The transcripts and field notes were double coded by (AA and DR) and the themes were identified independently by AA and DR. The themes were finalised by discussion with the whole research team. All findings have been drawn upon to inform the paper. The paper uses illustrative anonymised quotes throughout.

### Ethics

The study complied with the Code of Practice on Ethical Standards for Social Research Involving Human Participants operating in Public Health Sciences at Edinburgh University.

## Results

Several key themes emerged through the expert panel discussions. These related to: improving knowledge about SHS among carers and professionals, the goal and approach of future interventions, the complexity of the interventions, and issues around protecting children.

### Improving carers' and professionals' knowledge about SHS

The findings about flawed, confused or incomplete knowledge about SHS in the study sample resonated with participants' professional experience. Some panel participants talked about the parents, grandparents and communities that they worked with having become more aware of the health risks around SHS, but this was still limited. For example, grandparents talked of quitting smoking because they did not want to expose their grandchildren to their smoking, and smoking parents (especially mothers) were not taking their babies to bed with them, as this was now known to be a risk factor for sudden infant death. Panel participants expressed concern that interventions to reduce SHS in the home would be unlikely to be effective if the foundations and acceptance of the knowledge of the harm caused by second-hand smoke was not embedded in the lay population. There was therefore a need for more education about this.

Knowledge is such a major issue. In terms of initiatives, we are almost setting up initiatives when people don't have the knowledge about SHS, so we are one step ahead of ourselves. We are actually expecting people to make changes in the home, before they've got the knowledge that SHS is a real issue. (Expert 2)

I think we have got quite a bit to do about that the awareness about time and ventilation, the belief that air purifiers will do something. (Expert 7)

Credibility gap important. It is difficult to understand why a comparatively little amount of smoke can have such a large effect, when you look at what happens to smokers. CVD impact, because it is like that, but heavy passive smoking is like the equivalent of a light smoker. (Expert 11)

The panels thought that health and other professionals also had gaps in knowledge about SHS, including uncertainty about the risks, which could impact on both the priority given to addressing this issue and the likely effectiveness of interventions. In addition several panel participants had found that some health and other professionals and gatekeepers, including head teachers, were reluctant to promote smoke-free homes from fear of damaging their relationship with clients, including parents.

It is also about the individual health professionals because if we are going to reach out through midwives, through education or whatever, it is not just about the information that is going out to the public, it is about how we get that message out through health, education and social services. (Expert 6)

*Difficult messages for health workers because of a fear of being judgemental. Passive smoking a difficult argument to put forward*.

(Expert 12)

Given the gaps and confusion in knowledge about smoke-free homes, for both parents and professionals, the expert panel suggested that there needed to be to be an up-to-date review of the evidence base to inform education and training, particularly in relation to identifying key messages and how these could be incorporated into existing practice and new initiatives. Messages also needed to be clear and consistent, with coordination between national and local organisations. It was also thought important that these messages were tailored to the needs of individuals and target groups.

### Approaches to developing smoke-free homes- incremental or total ban

All panel participants agreed that the ultimate goal was smoke-free homes. However there was considerable discussion about the most feasible and effective way of achieving this. Panel participants argued that there was a need to develop a better understanding of what people can achieve, particularly those whose lives are shaped and constrained by social and environmental complexities and challenges. Several drew on their experiences of working with disadvantaged people, and communities, when discussing whether adopting a gradual or stepped approach towards smoke-free homes might be more realistic.

The gradual process bit is interesting, because respondent x and I have spoken about that. From the evidence base it should be a smoke-free home and that's what we should be trying to get towards, but this is so difficult. There is real value in using a staged approach, if people are saying it is gradual. It's like the high-rise flats, they can't physically go out of the house completely and it is trying to do a harm reduction route. (Expert 2)

This panel member also noted the resistance of health workers to situating the smoke-free message within the social context of people's lives and voiced concerns and that more complex messages might confuse people about whether there are safe levels of SHS.

We have had resistance from staff with this work, with the stepped approach. They feel that they are undermining the message they are selling by saying if they can't smoke entirely outwith the home, perhaps you can just smoke in one room. They think that is muddying the waters. (Expert 2)

Concerns were also expressed about the lack of an evidence base about the effectiveness of a stepped approach. It is not known whether partial restrictions eventually result in smoke-free homes, nor the most effective way of achieving this.

*If you modify the smoke-free home, are you compromising what you are wanting to ultimately achieve? Need to be careful of that too*. *(Expert 10)*

It is that question we need answered. If people start on this process, are they in a year's time more likely to have a smoke-free home than other people? Because if they are then the gradual process is acceptable, because it is taking them there. If they end up where they are not reducing risk, they are changing behaviour, but that behaviour change is not reducing risk in the longer-term, then that is a different situation. We don't have those answers yet, so we have to be fairly open. (Expert 5)

In addition panel participants were concerned that some of the strategies which study respondents used to deal with smoke in the home might not be part of a process of moving towards becoming smoke-free. Rather they could be more about being seen to do something or being able to continue to fit smoking around the performance of certain tasks, roles or use of spaces within the home.

.....candles for the aesthetics, and opening the window, I actually wonder how much they are just trying to justify that they are doing something? And actually they do know that there is still smoke in the room, but they are making an attempt to do something. (Expert 8)

Identified the fact when people selecting specific rooms to smoke in. Women smoke in the kitchen, because that is their space, their time out. That becomes a structured part of their day. (Expert 12)

However, given the everyday challenges of some people's lives participants argued that there needed to be a debate within the field as to whether the process of gradual change that some people and community initiatives have adopted should be supported, albeit while encouraging steps towards the ultimate goal of totally smoke-free homes. An additional argument put forward in support of accepting a gradual approach was that people who, motivated by concerns for their children, have created partial restrictions may find it disempowering to have this achievement negated or dismissed by a message that only a total restriction is useful.

### The complexity of the interventions

In addition to the complexities and challenges facing smoke-free interventions recognised above, the panels were very aware of the sensitivities and potential tensions around crossing the 'private' boundary of the home with 'public' smoke-free health promotion interventions. This discourse permeated all the discussions.

Some people will believe in a life choice of wanting to smoke whatever the thoughts are about the rights and wrongs of it. 'It is not because I don't believe it, it is because this is my house and this is want I have decided I am going to do'. (Expert 7)

Respectful of people's places, we would all be in danger of diving in and encouraging smoke-free homes and not thinking that it is their home, and space may or may not be limited, it is thinking about how to put over a message, rather than a blanket message. (Expert 10)

The panels discussed the importance of having a range of messages to promote smoke-free homes and that choice should be an essential component whatever the approach. However it was also argued that professionals needed to be 'much more straight' about giving information about SHS, for example, explaining how smoke travels in the home.

I think it is good having a variety because different people will latch onto different things. So if you are just promoting the one message then you lose people, but if you have got a raft of messages, something just clicks with some people. (Expert 8)

The panels were particularly aware of the reality of the everyday lives of many people such as responsibilities for children and lack of access to outside space. They thought that these realities would impinge upon the feasibility of the choices that people could make. There was concern that the stigmatising of smoking would create additional pressures for many and that the choice of a smoke-free home was not a real choice for many.

If you make people feel disapproved of, but actually you don't have another option, if the back of your tenement block is covered in glass and dog mess and your kids are three storeys up in a living room/kitchen, you are disapproved of, but what is your choice in that? Your choice is to do the wrong thing. (Expert 9)

### Protecting Children

The panels stated that protecting children from SHS is a growing public discourse, with protecting children from SHS in the home generally being seen as more important than protecting adults. Panel participants also agreed that the significant illnesses experienced by children of smokers contribute to, and are further exacerbated by, existing inequalities and can impact upon the life chances of many children. Other themes emerged related to children's levels of agency and power in reducing their exposure to SHS in the home, concepts of the vulnerable child, the role of carers and professionals (health and others) in protecting children and the rights of children compared to adults.

The potential for the voice of children to be central in creating smoke-free homes was acknowledged.

Is some of the behaviour change in grandparents as a result of information that is coming from the grandchildren, they are getting information about passive smoking and taking that back to grandparents, and that is making them change their behaviour? In my experience it is often children who bring grandparents or parents over to the smoke-free home stand and say 'see you should be taking note of this'. (Expert 5)

Children were recognised as potential agents of change. But panel participants also voiced concerns about the ethical issues of children not always having the power to change their harmful living environment whilst being educated about the harmful effects of exposure to SHS.

What would you do with children who actually can't modify their circumstances? People were worried about what the reaction in children would be, and certain feedback from the schools indicated that it hasn't been a major issue. On the other hand we don't really know what goes on. But are there ethical issues in how you deal with that? (Expert 11)

Also one panel member reflected upon the situation for those children who live in communities where smoking is highly visible and consider smoking to be the norm.

When we did the consultation on 'Towards a Future Without Tobacco', the young people in deprived areas didn't believe the national smoking statistics because they live in communities where everybody is smoking. So they think it is the norm and that is a huge thing. If you live in a community, there is a certain rate of smoking, you are more likely to make some changes. If you live in a community where everybody is smoking, it is that much harder to make those shifts. (Expert 5)

Discussions also focussed on the extent to which children's exposure to SHS was viewed as harmful and therefore should be treated as a child protection issue. This was regarded as a complex area, reflecting different definitions and concepts of harm and vulnerability by statutory child protection services and individuals. These to some extent were seen as relating to different disciplines and agencies' priorities and boundaries.

We think vulnerable as being all the reasons why people access services. Seen as vulnerable because they are children, it's an age issue. Language of 'vulnerable' may be problematic. We were not asking for kids to be taken into care, but just to up the ante about the effects of passive smoking. And to see tobacco as a drug of harm and to help education and awareness raising. (Expert 7)

However, panel participants thought that there was an increasing awareness that 'looked after' children should be in smoke-free homes.

Starting to look at it more with children in foster care, looked after accommodated. A child could come back and claim the local authority did not look after me because I have been damaged through smoking in the home. (Expert 2)

The views about protecting sick children from SHS were more robust and appeared to part of a changing discourse of protection for sick children.

First, you would ask them, whereas in the past you maybe would not have done. I would quite strongly say smoking and asthma don't mix. I can't say you must stop smoking, in the sense that they won't necessarily stop, but I make it very clear than this is not helping and that it is actually quite dangerous. I think people do know about asthma increasingly. (Expert 8)

## Discussion

The panels recognised that there is a growing risk discourse of protecting children from SHS within their homes. While there is some evidence that media campaigns might be effective in increasing restrictions on smoking in the home [10,31], there is little current evidence on the relative effectiveness of specific interventions in the setting of the home to achieve this, particularly for local community and individual approaches. The questions posed to the expert panel were presented in an open way and aimed to elicit responses about both population and individual/community level interventions. However, a limitation of the study is that the expert panels' responses to the questions were mainly located within an individual and local community focus. The panel, despite including those who worked at the national level, considered the national perspective only in terms of support for national training and for guidance on clarifying the focus of the smoke-free home message, rather than considering national media campaigns targeting the whole population. This may have resulted from the panels mainly reacting to the results of the primary study. These findings should therefore be considered within this restricted individual and community focus. In addition the expert panels, whilst chosen for their expertise from across one country to represent local and national perspectives, were a small purposive rather than a representative sample, and thus these findings should be treated with appropriate caution.

This paper aimed to provide an account of the views of people working in tobacco control and health promotion in order to illuminate the issues and debates around achieving smoke-free homes. It also provides a nuanced understanding of the complexity of people's daily lives and the barriers that can be experienced, by both professionals and parents, in trying to implement smoke-free homes. And in particular, those barriers, that are further exacerbated by the low socio-economic status of parents that were identified by the panels. [24-27]. It is suggested that the effectiveness of interventions by health professionals and others, who work with children and parents, may be enhanced by an understanding of the complexity of these debates inherent in the policy and practice of smoke-free home interventions.

The expert panels made a number of suggestions for developing smoke-free homes' interventions. First, improvements in health professionals' knowledge of the effects of SHS may provide a foundation to improve successful local/community interventions. The findings suggested that health professionals could be more transparent about the harm that SHS can cause children, and particular attention should be paid to those children who are medically at risk from SHS. There is a need for continuing professional training and education on SHS that also supports the development of appropriate attitudes and skills for professionals to work effectively with smokers on this issue. Clearly the restrictions advocated by these expert health professionals need to be located within an understanding of the constraints posed by the everyday realities of people's lives. Indeed our findings, drawn from the primary study, suggested that some parents may feel unable to achieve a totally smoke-free home and many may adopt strategies that may be ineffective. However, the authors suggest that valuing the motivation of parents to introduce some form of restriction may be important in ensuring that they are not further disempowered. Furthermore an assessment of the perceived barriers experienced by some parents is critical in achieving the goal of smoke-free homes. The experts were uncomfortable about the lack of clarity in such processes, but they were also able to situate people within their relevant life contexts and support parents through an approach that recognises the environment of their health decisions.

Secondly the expert panels suggested that there is an urgent need to develop a more robust evidence base of the effectiveness of different individual/community level approaches to smoke-free homes, not least the relative effectiveness of staged/incremental compared to total ban approaches [31]. Some, including several of our panel members, have argued that a staged approach may be more realistic and sustainable in ultimately protecting children [18], whilst still maintaining the goal of smoke-free homes. Underpinning this approach is the principle, advocated by the panel, that any attempt to become smoke-free and to protect children from SHS should be valued and that such attempts should not be negated by goals that seem not immediately feasible for some parents. In particular they argue that mothers should not be further disempowered by the stigma of their smoking and the constraints they may experience in creating smoke-free homes. Others in the literature have considered the ineffectiveness of harm reducing strategies and advocated for the clarity and unambiguous message of totally smoke-free homes [6].

We, the authors, would argue that a clear goal of smoke-free homes should be advocated but that this approach should be located within tobacco control practice that is both sensitive to inequalities and gendered lives [32-34]. It is important for these debates to be further explored and for public health strategies to be developed that are not disempowering or stigmatising of parents, particularly low income parents [11,32] but at the same time we argue for a sensitive consideration of the rights of the child to a healthy environment, particularly the sick child.

Third, the expert panels advocated that the concept of protecting children's rights needs to be sensitively located within an understanding of protecting mothers from processes that will be disempowering, particularly those mothers who may have little power or scope to make the 'healthy choices' [33] Furthermore the panels concluded that sensitivity towards the smoker who may be disadvantaged and already disempowered versus the rights of the child needs to be incorporated into a contextual understanding of people's everyday lives.

We would emphasise that this is an extremely challenging and sensitive area of public health. But importantly the growing discourse about protecting children and expressed motivation by parents and other carers to protect children is encouraging. The experts in the study were not in favour of formal child protection measures. However, they did want child welfare agencies to be more direct and/or assertive in educating parents about the harmfulness of SHS exposure, particularly among 'medically at risk' children such as those with asthma.

In summary SHS constitutes a serious public health problem for children but it is also a problem imbued with complexity that poses ethical problems for public health. On the one hand the home is a private space and there is some resistance found in the ethical debates inherent in public health literature to the blurring of the public/private boundary for smoke-free public health interventions [35]. This is often articulated by libertarian arguments advocating the rights of smokers in their own home and opposing perceived encroachment of the State into private space. On the other hand the rights of the child are enshrined in the UN Convention on the Rights of the Child [36,37]. The Convention charges the community to ensure that the best interests of the child are always considered and protected. Despite the accepted evidence linking SHS to the cause and exacerbation of a number of childhood illnesses [10], many children often do not have the personal power to protect themselves from exposure to SHS [11,12]. Such sensitivities around the public/private boundary may not be applicable in all cultural contexts. However one of the aims of the paper is to bring the discourse of protecting children in the home into wider tobacco control discourses globally and we have attempted to explore these tensions.

## Conclusion

In conclusion, this paper has not resolved the tensions inherent in smoke-free homes interventions, but has attempted to provide a rich contextual understanding of the issues; that is embedded within the expertise of those involved in tobacco control and health promotion. Importantly it is their understanding of the reality of people's lives and the scope parents have to make changes in their lives that will inform a sensitive and empowering approach towards smoke-free homes.

## Competing interests

The authors declare that they have no competing interests.

## Authors' contributions

AA, SC-B, CM and DR designed the study. DR wrote the first and subsequent drafts of the paper. RP undertook most of the interviews in the qualitative study and organised the expert groups. DR and AA facilitated the groups and analysed the findings. All authors read and approved the final manuscript.

## Pre-publication history

The pre-publication history for this paper can be accessed here:


